# Effects of Virtual Reality–Based Interventions on Preoperative Anxiety in Patients Undergoing Elective Surgery With Anesthesia: Systematic Review and Meta-Analysis

**DOI:** 10.2196/55291

**Published:** 2025-04-30

**Authors:** Huiyuan Li, Pak Lung Chiu, Defi Efendi, Haiying Huang, Ka Yan Ko, Cho Lee Wong

**Affiliations:** 1 The Nethersole School of Nursing Faculty of Medicine Chinese University of Hong Kong Hong Kong China (Hong Kong); 2 School of Nursing Fudan University Shanghai China; 3 Department of Pediatric Nursing Faculty of Nursing Universitas Indonesia Depok Indonesia; 4 Neonatal Intensive Care Unit Universitas Indonesia Hospital Depok Indonesia; 5 Department of Pediatric Hematology/Oncology Guangzhou Women and Children Medical Center Guangzhou China; 6 School of Nursing Hong Kong Polytechnic University Hong Kong China (Hong Kong)

**Keywords:** meta-analysis, preoperative anxiety, surgery, systematic review, virtual reality, anesthesia, exposure approach

## Abstract

**Background:**

Preoperative anxiety is a common yet often neglected problem for patients undergoing surgery. Virtual reality (VR)–based intervention is a promising alternative with benefits for managing preoperative anxiety. However, the components of VR-based intervention and its effectiveness on preoperative anxiety in patients undergoing elective surgery with anesthesia remain unclear.

**Objective:**

This study aimed to identify the major components (ie, device, medium, format, and duration) of VR-based interventions and summarize evidence regarding their effectiveness in reducing preoperative anxiety in patients undergoing elective surgery with anesthesia.

**Methods:**

Allied and Complementary Medicine, Chinese University of Hong Kong Full Text Journals, CINAHL via EBSCOhost, Cochrane Library, Joanna Briggs Institute EBP Database, EMBASE, MEDLINE via OvidSP, PubMed, PsychINFO, Scopus, China Journal Net, and WanFang Data Chinese Dissertations Database were searched from inception to February 2025. Randomized controlled trials (RCTs) of VR-based interventions for patients undergoing elective surgery with anesthesia were included. The Cochrane Collaboration’s tool was used for risk of bias assessment. A random effect model was used for pooling the results.

**Results:**

A total of 35 RCTs with 3341 patients (female: n=1474, 44.1%) were included in this review, of which 29 RCTs were included for meta-analysis. Compared with usual care, VR-based interventions showed substantial benefits in decreasing preoperative anxiety in patients undergoing elective surgery (standardized mean difference [SMD] 0.65, 95% CI 0.37-0.92; *P*<.001). Regarding the subgroup analysis, VR-based intervention showed significant but moderate effects on preoperative anxiety in the pediatric population (SMD 0.77, 95% CI 0.32-1.22; *P*<.001) compared to the adult population (SMD 0.58, 95% CI 0.23-0.93; *P*=.001). The distraction approach showed more significant effects (SMD 0.73, 95% CI 0.24-1.21; *P*=.004) on preoperative anxiety than the exposure approach (SMD 0.61, 95% CI 0.27-0.95; *P*<.001).

**Conclusions:**

Patients undergoing elective surgery with anesthesia may benefit from VR as a novel alternative to reduce preoperative anxiety, especially pediatric patients via the distraction approach. However, more rigorous research is needed to confirm VR’s effectiveness.

## Introduction

Preoperative anxiety comprises subjective emotional, cognitive, and physiological responses triggered by the stressful event of surgery [1]. The incidence of preoperative anxiety is estimated to range from 11% to 80% [2,3]. The preoperative anxiety experienced by presurgical patients is usually induced by concerns about general health and uncertainty regarding the outcomes of the surgery, the type of surgery and anesthesia, pain and discomfort after the surgery, the feeling of helplessness, loss of independence, and fear of mortality [4]. Pediatric patients also experience preoperative anxiety due to separating from their parents and fear of the unknown, and they manifest their preoperative anxiety in many ways, including crying, attempting to escape from the medical health care professionals, and refusing surgery [5]. Preoperative anxiety induces hypertension and increases the heart rate [6]. The increase of adrenaline, norepinephrine, and plasma cortisol caused by preoperative stress suppresses the patients’ immunological responses, making them vulnerable to diseases and other postoperative complications, such as nausea, vomiting, respiratory distress, and heart attack [7,8], influencing postoperative recovery and treatment satisfaction [9,10]. These detrimental impacts underscore the urgent need for effective interventions to reduce preoperative anxiety.

Pharmacological interventions, such as administering sedatives and antianxiety drugs, have been demonstrated as not the best solution for managing preoperative anxiety [11,12], and they could result in negative consequences [11,13,14]. Studies have suggested that distraction [15], clown therapy [16], handheld video games [17], and audio-visual interventions [18] could effectively reduce preoperative anxiety in children. However, there is limited evidence regarding nonpharmacological alternative interventions in the adult population. Furthermore, music therapy has shown its efficacy on preoperative anxiety [19], however, the requirements for specialized training and potential risks of increasing the workload of health care professionals limit its generalizability and replicability. Additionally, traditional educational interventions have had controversial results [20,21], as traditional interventions may be dependent on variability in individual responses, and they thus may not be sufficient for patients with severe anxiety [22,23]. Considering the limitations, it is imperative to consider alternative means to effectively manage preoperative anxiety in this population.

Virtual reality (VR) is a digital simulation of a computer-generated situation or environment where orientation and 3D interaction are possible [24] by means of extremely sophisticated interfaces. When applied in patients undergoing elective surgery, VR technology may have the ability to modulate subjective experience during the perioperative periods, where it may be used to offer respite from stressful or confining environments, such as hospital wards or surgical departments, or as a distraction from chronic or procedural pain or anxiety [22,25]. The VR-based interventions used 2 main approaches for managing anxiety: the distraction approach and the exposure approach. The distraction approach assumes that a limited amount of information can be processed at a time by an individual. As the VR creates a distraction by predominantly recruiting the individual to a specific attention task, their attention to anxiety can be restricted [26-28]. Meanwhile, the exposure approach is effective in managing anxiety-inducing conditions by exposing the individual to virtual experiences of the distressing environment prior to real-life exposure to the environment so that avoidance and resistance to the anxiety-inducing environment can be reduced [29-33]. Although VR exposure has shown promise for preparing patients for anxiety-provoking medical procedures [34], the evidence of its potential benefits as a preparatory tool compared to other mediums in specific medical procedures (ie, preoperative anxiety during perioperative periods) requires further investigation [35].

Several systematic reviews and meta-analyses [22,36-38] have been conducted, and the results suggested positive results in managing preoperative anxiety by providing information and exposing patients to the virtual environment of the operating theatre or providing distractive intervention through VR technology. However, all these reviews were conducted with adolescents and children, which may limit the generalizability of the results on different age group populations. Koo et al [39] conducted a systematic review and demonstrated the effects of VR on preoperative anxiety in the pediatric and adult population. However, the necessary components of VR intervention development (ie, VR device, VR intervention procedure, duration, medium, and control group contents) were not systematically summarized, and the efficacy of VR intervention via different mediums (ie, exposure or distraction) remains unclear. Another updated systematic review illustrated the efficacy of VR in the adult population [40], but a systematic synthesis of the components of VR is still lacking. Although the effects of VR mediums (ie, VR distraction and VR exposure) were examined in adults; to minimize intervention heterogeneity, this study only analyzed the 2 VR interventions separately without using subgroup analysis for comparison. Additionally, the effect of VR on different age groups (ie, children vs adults) remains to be clarified. Thus, summarizing the current evidence is imperative for adopting this novel intervention to address this prevailing problem in surgical patients in a local setting.

This review aimed to identify the effects of VR-based interventions on reducing preoperative anxiety in patients undergoing elective surgery with anesthesia and identify the major components (ie, device, medium, content, format, and duration) of VR-based interventions for this population.

## Methods

The PRISMA (Preferred Reporting Items for Systematic Reviews and Meta-Analyses) guidelines [41] were adopted for this review for problem identification, literature search, data analysis, and evaluation and summary of the results (Multimedia Appendix 1).

### Search Strategy

An exhaustive search was conducted on 11 electronic databases, including Allied and Complementary Medicine, Chinese University of Hong Kong Full Text Journals, CINAHL Complete via EBSCOhost, Cochrane Library, Joanna Briggs Institute EBP Database, EMBASE, MEDLINE via OvidSP, PubMed, PsychINFO, Scopus, China Journal Net, and WanFang Data Chinese Dissertations Database from their inception until February 2025. The search was further supplemented with bibliographies, Google searches, and manual searches of reference lists of relevant or similar studies to extend the search areas. The publication languages were restricted to English, traditional Chinese, or simplified Chinese. We aimed to ensure a globally representative literature review by including diverse databases, particularly those focusing on Asian research (ie, Chinese University of Hong Kong Full Text Journals, China Journal Net, and WanFang Data). We applied strict inclusion criteria to select high-quality studies, aiming to highlight diverse perspectives on VR interventions in the context of cultural diversity. We intentionally included dissertation databases to gain insights into preliminary research and innovative interventions not yet published in peer-reviewed literature. The searching strategy with search terms is presented in Multimedia Appendix 2.

### Eligibility Criteria

#### Inclusion Criteria

Inclusion criteria were as follows: (1) randomized controlled trials (RCTs), (2) patients undergoing anesthesia for elective surgery, (3) studies investigating the effects of VR-based interventions via a head-mounted display (HMD) with either fully immersive 3D computer-generated environments or 360° videos in surround stereoscopic vision, (4) the comparison group could be usual care or other interventions, and (5) preoperative anxiety was investigated either as a primary or a secondary outcome.

#### Exclusion Criteria

The following types of studies were excluded: (1) protocol studies, conference proceedings, reviews, or abstracts; (2) nonpreoperative interventions, such as intraoperative and postoperative interventions; (3) simulation interventions, such as interventions with visual and audio stimulation but no interaction between the user and the computer-generated world; and (4) nonclinical settings, such as in a simulation laboratory setting and studies with participants who would not undergo surgery with anesthesia.

### Screening

Two researchers (HL and PLC) independently screened titles and abstracts of retrieved studies and deleted irrelevant and duplicated studies. Full texts of the potential studies were then screened for eligibility by the 2 researchers (HL and PLC). A third researcher (CLW) resolved any disagreement.

### Data Extraction

The data extracted from each eligible study included author, year of publication, the origin of study, type of surgery, sample size, sex, the mean age of the population, VR devices, the approach used in VR, intervention characteristics (ie, duration), control, measurement tool for preoperative anxiety, and key findings using a standardized data extraction form.

### Quality Assessment

The quality of the included studies was evaluated by 2 independent appraisers (PLC and CLW) using the Cochrane Risk of Bias tool for assessing the risk of bias [42], in which the methodological quality of the trials for randomization sequence generation, allocation concealment, blinding of participants and personnel, blinding of outcome assessment, incomplete outcome data, selective reporting, and other biases were evaluated as either “low risk,” “high risk,” or “unclear.” Any disagreements were resolved by a third researcher (HL).

### Statistical Analysis

The meta-analysis was conducted to pool data of the same outcome measured in 3 or more RCTs using Review Manager (version 5.4; The Cochrane Collaboration) for MacOS. Otherwise, a narrative synthesis was conducted. A random effect model was used for pooling the results and showing conservative summary effect estimates [43]. The mean score and SD for preoperative anxiety in the included studies were extracted from them. As different measurement tools were used in the included studies, the effect size for preoperative anxiety was analyzed using the standardized mean difference (SMD) and 95% CIs. Meanwhile, the means and SD of 3 studies [24,44,45] were calculated from median values and IQRs [46]. A positive effect size was adopted if the VR-based interventions reduced more preoperative anxiety than the control. Other nonpreoperative anxiety outcomes, such as satisfaction scores and behavioral outcomes, were not extracted for the meta-analysis. Heterogeneity was evaluated using the Cochran *Q* test and the *I*^2^ statistic. A Cochran *Q* test result with a *P*<.10 means statistically significant heterogeneity. The extent of heterogeneity was assessed through *I*^2^ statistics, and 75%, 50%, 25%, and 0% indicated high, moderate, low, or no heterogeneity, respectively [47]. In this review, subgroups were formed according to the type of population (adult or children) and intervention approach (distraction or exposure).

## Results

### Overview

**Figure 1:**
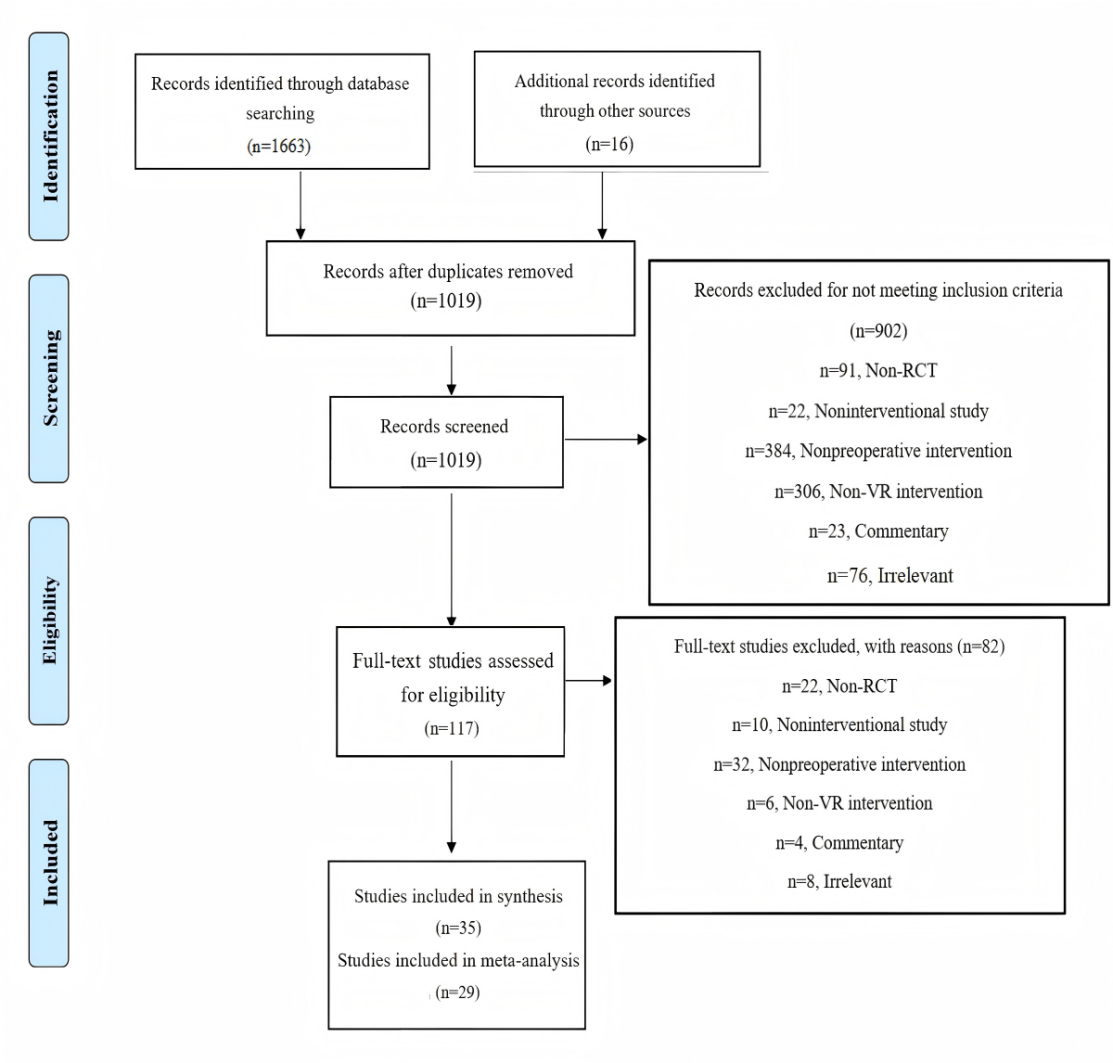
PRISMA 2009 flow diagram of the literature search process. PRISMA: Preferred Reporting Items for Systematic Reviews and Meta-Analyses; RCT: randomized controlled trial; VR: virtual reality.

The combined search yielded 1679 records. Duplicate records were excluded during the initial screening, which yielded 1019 studies for subsequent screening. Based on the information from their titles and abstracts, 902 studies were excluded, and 117 studies were retrieved in full-text format to assess their compliance with the inclusion and exclusion criteria. A total of 82 studies were excluded after full-text screening because of non-RCT studies (n=22), noninterventional studies (n=10), nonoperative intervention studies (n=32), non-VR intervention studies (n=6), commentaries (n=4), and irrelevant studies (n=8). Subsequently, a total of 35 studies were included for quality appraisal. A total of 29 studies were included for data analysis, of which 5 studies were excluded because of incomplete data. Although Chinse databases and dissertation databases were searched, no eligible Chinese-language studies or dissertations were ultimately identified for inclusion in this review. The search process is presented in Figure 1.

### Risk of Bias

**Figure 2:**
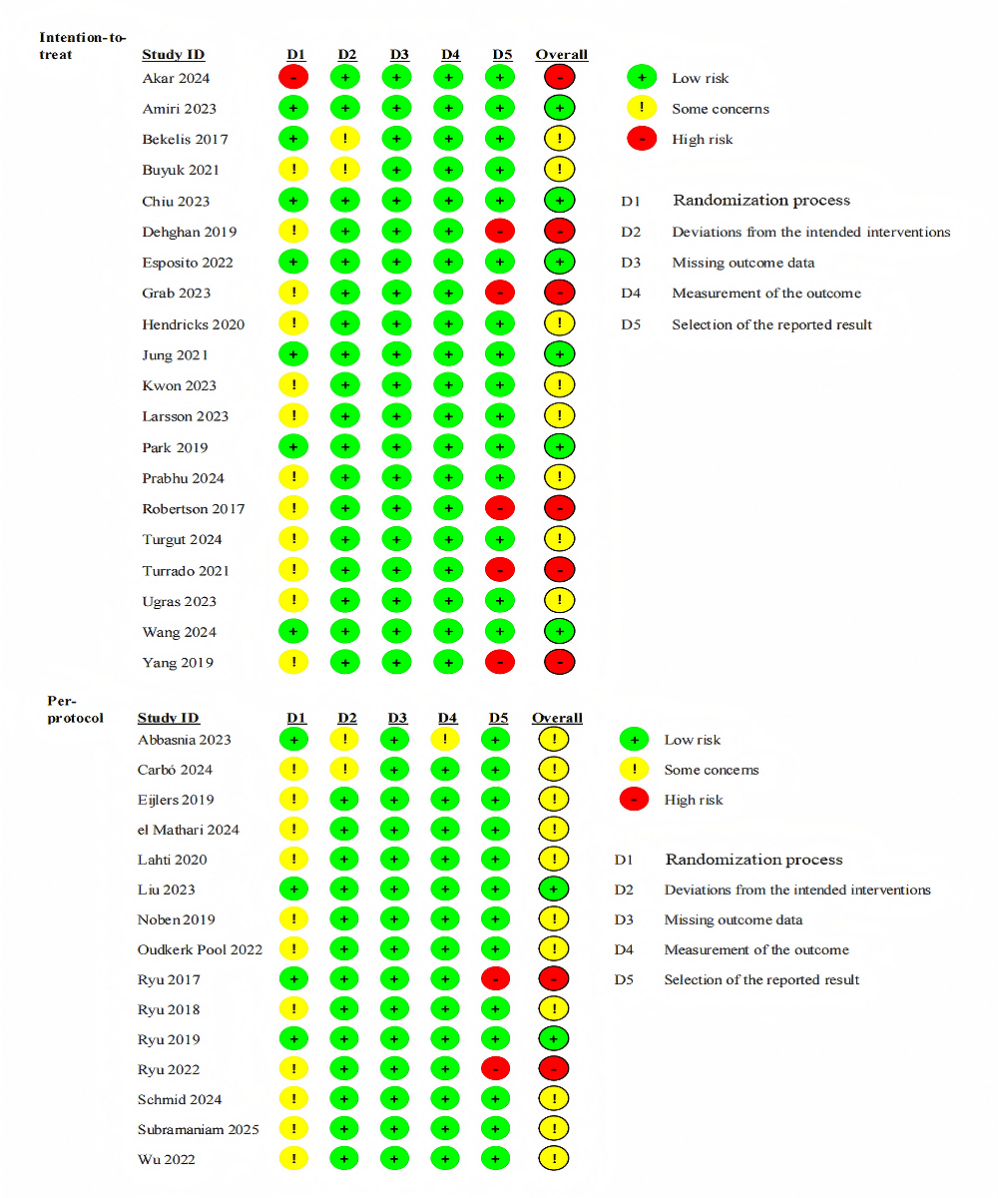
Summary of risk of bias of included studies. [24,30,44,45,48-78].

Figure 2 summarizes the risk of bias in the included studies. The overall quality of evidence of the studies included was moderate due to the randomization process and concealed allocation. Eight studies were assessed to have a high risk of bias due to the randomization process [48] and the selection of reported results [30,49-54]. Noben et al [55] did not provide information on the blinding of outcome assessment and the potential threat of detection bias cannot be mitigated. In addition, research participants cannot be blinded to most VR-based interventions, so they are aware of the study group assignment.

**Figure 3:**
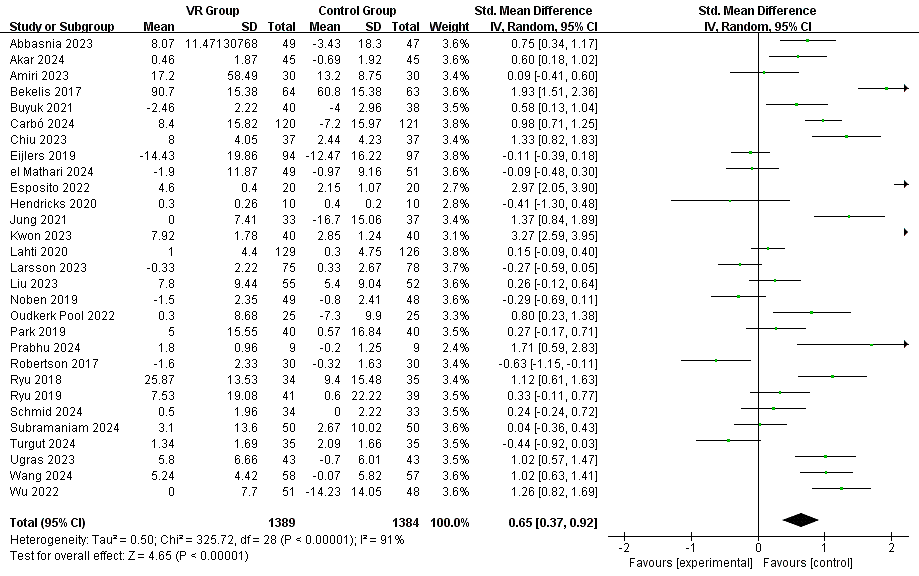
Random effect meta-analysis for the effect of VR-based intervention on preoperative anxiety. VR: virtual reality. [24,44,45,48,51,55-78].

### Characteristics of the Studies

Multimedia Appendix 3 [24,30,44,45,48-78] presents the characteristics of the studies. The 35 included RCTs were conducted between 2017 and 2025 and originated from Australia (n=2) [51,56], China (n=3) [57-59], Finland (n=1) [60], France (n=1) [61], Germany (n=1) [50], Hong Kong (n=1) [62], Iran (n=3) [30,63,64], Italy (n=1) [65], Korea (n=7) [44,45,49,53,54,66,67], the Netherlands (n=4) [24,55,68,69], Spain (n=2) [52,70], Turkey (n=4) [48,71-73], and the United States (n=5) [74-78]. All studies were published in English.

### Characteristics of the Participants

A total of 3341 participants were included in this review (female: n=1474, 44.1%). The sample size of recruited participants in each study ranged from 20 to 255. A total of 13 studies included a total of 1163 child participants (younger than 18 years), and 22 studies had a total of 2178 adult participants. A total of 13 studies involved patients undergoing general elective surgery [44,45,49,54,56,59,62,65-67,70,72,77], and the other 12 studies involved patients undergoing abdominal (n=6) [30,52,55,58,63,73] and heart surgery (n=6) [48,50,61,64,68,69]. Another 10 studies involved patients with diverse surgery types, including knee surgery (n=3) [51,53,77], dental surgery (n=2) [24,60], thoracic surgery (n=2) [25,78], cranial and spine procedures (n=1) [74], vascular surgery (n=1) [57], and circumcision (n=1) [57]. Detailed characteristics of participants of the eligible studies are shown in Multimedia Appendix 3 [24,30,44,45,48-78].

### Characteristics of the Interventions

#### Devices Used in the VR Interventions

Studies used a variety of VR devices. A total of 23 studies used computer-connected VR HMDs with built-in display units. Most studies adopted the Oculus series VR devices (n=9; ie, Oculus Rift [44], Oculus Go [54,56,69,72], Oculus Quest 2 [50,58,62], and unclear Oculus series [74]), followed by using the unmentioned brand HMD (n= 4) [30,61,65,78], the HTC Vive HMD (n=3) [24,53,77], and PICO G2 (n=2) [66,68]. The other 12 studies that used VR HMDs required additional smartphone devices as display units, including the Samsung Gear VR device (n=8) [45,48,49,51,60,70,75,76] and an unmentioned brand of VR eyeglasses (n=4) [55,63,64,71].

#### Approaches and Content of VR Interventions

In total, 10 studies adopted the distraction approach of VR interventions by distracting adult participants using virtual landscapes [48,51,58,60,61,63,73,75,77,78], while 3 studies adopted the distraction approach in children [65,71,76]. A total of 22 studies adopted the exposure approach of VR interventions: 10 studies [24,30,44,45,49,54,59,67,70,72] exposed child participants to the operating theatre environment with a VR tour, while 12 studies [50,52,53,55-57,62,64,66,68,69,74] exposed adult participants to information related to surgeries.

The storyline developed in the virtual environment varies in the included studies. Among the 13 studies that adopted the distraction approach, 3 studies adopted 2 relaxing VR environments (ie, walking in the forest and water skiing) [71], a 5-minute movie with 3D interaction [65], and gaming with an animated animal [76] for children. Regarding adult populations, 6 studies contained different optional landscapes and nature scenes with natural sounds [58,61,63,78] or relaxing music [60,73]. Three studies created a single nature scenario with natural sounds [48,77] or a narrated progressive muscle relaxation technique [51]. Only Hendricks et al [75] developed a nonviolent game in which patients could move their heads and visual gazes to target objects in an energetic cartoon world.

A total of 22 studies described the virtual environmental storyline using the exposure approach. For child populations, 3 studies simply described the virtual environment developed in their studies, which included preoperative and postoperative experience for the day of the surgery [72], steps of going to the operating room [30], and a VR-guided tour of the operating theater [49]. The other 7 studies explained the detailed preoperative preparation process to children in a friendly manner. The storyline began in the holding area after admission, then transported into the corridor to the operating theatre, during the operating room, and ended in the recovery room [24,44,45,54,59,67,70]. Among these, 2 studies developed 2 versions for children of 2 different age ranges to attune explanations to a child’s developmental level [24,70]. Three studies created a cartoon penguin, acting as a pediatric patient, to introduce and explain the perioperative preparation process [45,54,67]. Ryu et al [44] and Wu et al [59] explained the preoperative process and general anesthesia induction via a game and an adventure story, respectively.

As for adult populations, only 1 study simply described the virtual environment developed in the study [74], the other 7 studies explained detailed storylines simulating the entire journey of the perioperative process, including comprehensive elements of the real-world environment at the hospital, featuring the preoperative admission suite, preanesthetic bay, operating theatre, postoperative recovery room, and medical staff [52,56,62,64,66,68,69]. Another 4 studies provided additional descriptions of the virtual environment in the storyline. Noben et al [55] provided comprehensive VR videos for women undergoing cesarean delivery, including admission to the ward, the operating room, placement of spinal analgesia, and to the birth of the baby when the gynecologist lifts the baby above the sterile environment. Besides a preoperative VR experience for patients expected to undergo elective arthroscopic knee surgery, a virtual environment describing the anatomy of the knee, as well as their own lesion of interest in need of arthroscopic procedure was provided by Yang et al [53]. Grab et al [50] also developed a VR app allowing the selection of a specific surgical procedure subsequently loading the respective presentation to the user’s view. Additionally, Liu et al [57] created a VR video consisting of 3 parts, including an introduction to the operation room, a patient interview, and a scenic tour.

#### Duration of the VR Interventions

Among the 13 included studies adopting a distraction approach, except that unspecified duration was adopted by 3 studies [48,60,76], the average duration of the other 10 studies was 10.85 minutes, ranging from 4.5 [71] to 20 minutes [61,75]. Of the 22 studies implementing exposure approach interventions, only 3 studies [53,62,72] did not specify the duration; the other 19 studies had an average duration of 8.22 minutes, ranging from 3 [56] to 21.60 [50] minutes.

### Characteristics of the Control Groups

All participants in the control groups received care as usual in the included 35 studies. “Care as usual” in 24 studies referred to providing standard preoperative information. Nine studies used various mediums to provide preoperative information, such as video (n=6) [51,64,67,74,77,78], 3D printed models (n=1) [50], tablet-based games (n=1) [75], and multiple mediums (n=1) [57]. The care as usual in another 2 studies referred to parents of patients touching and caring for their children [30] or no intervention [60].

### Characteristics of the Outcomes

Different measurement tools for preoperative anxiety were used among the included 35 studies. For the 13 studies conducted in children, most studies adopted either the Yale Preoperative Anxiety Scale [30] or the modified Yale Preoperative Anxiety Scale (n=9) [24,44,45,49,54,59,67,70,76]. For studies conducted in adult populations, the commonly used tools included the State-Trait Anxiety Inventory (n=9) [50,52,57,63,64,68,75,77,78], the Amsterdam Preoperative Anxiety and Information Scale (n=6) [53,62,66,68,69,74], Visual Analogue Scale (n=6) [48,50,55,56,61,77], and Hospital Anxiety and Depression Scale (n=3) [51,52,58]. Four studies adopted 2 different scales to evaluate preoperative anxiety in adults [50,52,68,77].

Apart from measuring preoperative anxiety, the included studies also assessed the impact of VR-based interventions on other outcomes, including physiological indicators, physical symptoms, psychological symptoms, behavioral problems, and other outcomes. Physiological parameters like pulse [65], respiratory rate [77], systolic and diastolic blood pressure [51], and galvanic skin response [51] were assessed in 4 studies. Physical symptoms evaluated in the included studies were pain (n=10) [24,53,58,59,62,63,70-72,77], sleep quality (n=2) [57,58], and postoperative complications (n=5) [24,58,59,70,75]. The commonly assessed psychological symptoms included fear (n=2) [48,71], stress (n=5) [53,55,62,65,74], satisfaction (n=14) [44,50,53,57,59,61,62,66-68,70,72,74,76], preparedness (n=3) [53,62,74], and self-efficacy [57]. Behavioral (n=6) [24,44,45,49,59,77] and other outcomes (ie, length of hospital stay [62,75]) were also evaluated.

The assessment time points varied among the included studies. Most studies evaluated preoperative anxiety at pre- and postintervention (n=29) [30, 44, 45, 49-55, 57, 58, 60, 61, 63-75, 77, 78]. Another 6 studies evaluated preoperative anxiety at 3 time points before surgery (n=1) [62], and before (n=2) [48,56] or during (n=3) [24,53,76] induction of anesthesia.

### Effects of the VR-Based Interventions on Preoperative Anxiety

**Figure 4:**
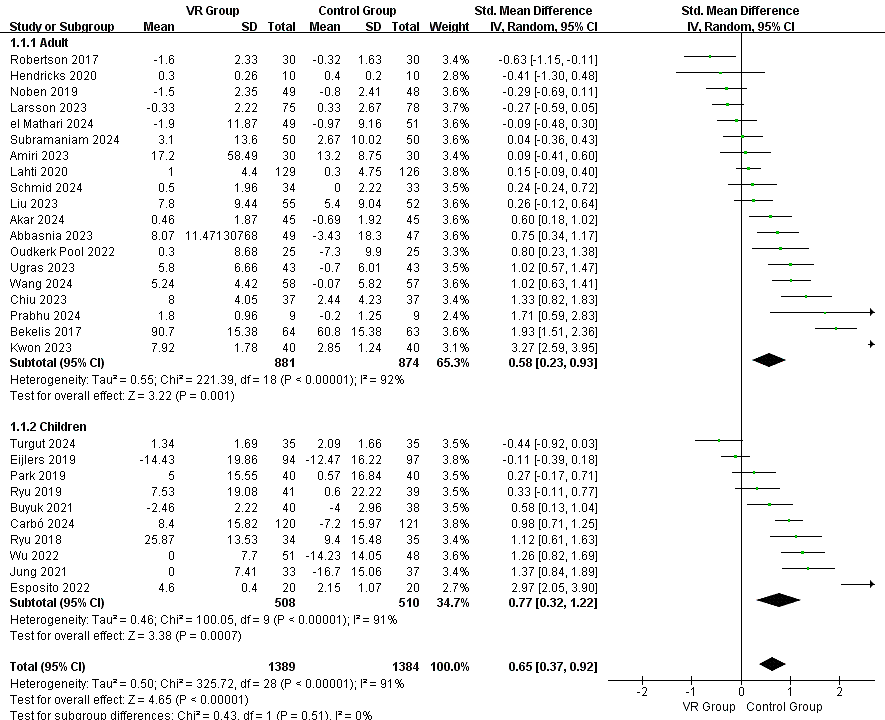
Random effect meta-analysis for the effect of intervention on adults and children [24,44,45,48,51,55-78]. VR: virtual reality.

Figure 3 summarizes the effects of VR-based intervention on preoperative anxiety involving 29 RCTs. According to the meta-analysis shown in Figure 4, the overall pooled effect size for both the adult and child populations was moderate (SMD 0.65, 95% CI 0.37-0.92; *P*<.001), indicating the substantial benefit of VR-based interventions compared to the usual care experienced by the control group, but with considerable heterogeneity (*I*^2^=91%; *P*<.001).

Among the adult population, the pooled effect size was also moderate, which indicated the beneficial effect of the VR-based interventions (SMD 0.58, 95% CI 0.23-0.93; *P*=.001; *I*^2^=92%). With regard to the child population, the pooled effect size was medium (SMD 0.77, 95% CI 0.32-1.22; *P*<.001; *I*^2^=91%), suggesting the significant effects of the VR-based interventions compared to care as usual (Figure 4).

**Figure 5:**
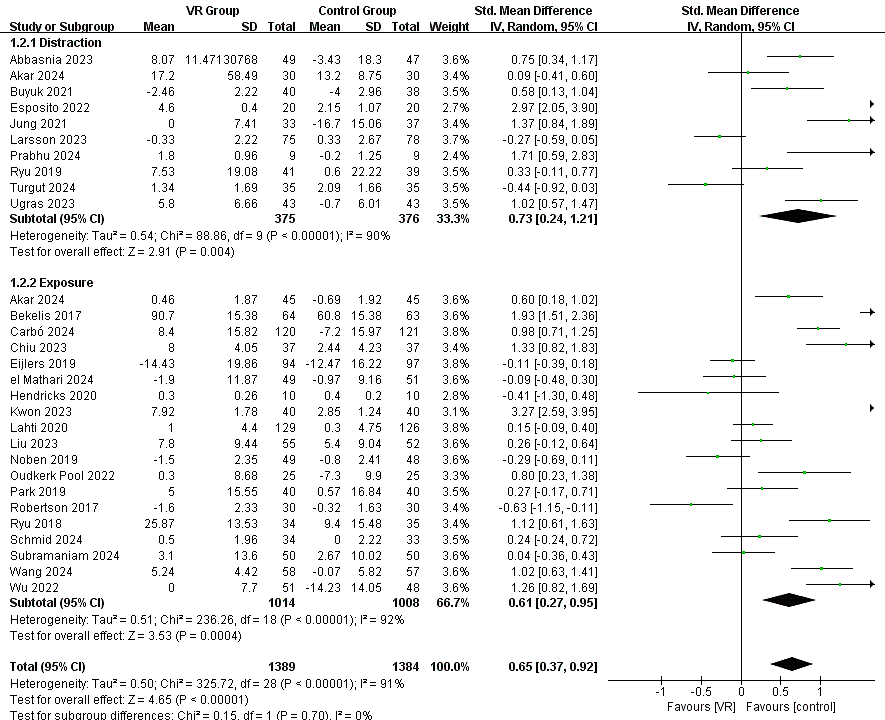
Random effect meta-analysis for the effect of interventions using the distraction and exposure approaches [24,44,45,48,51,55-78]. VR: virtual reality.

According to the subgroup analysis of the intervention approaches depicted in Figure 5, the distraction approach showed a more significant effect (SMD 0.73, 95% CI 0.24-1.21; *P*=.004; *I*^2^=90%) than the exposure approach (SMD 0.61, 95% CI 0.27-0.95; *P*<.001; *I*^2^=92%).

## Discussion

### Principal Findings

To the best of our knowledge, this is one of the few meta-analyses to explore the effectiveness of VR to reduce anxiety in adult and pediatric populations simultaneously. We used an extensive search process of 11 databases and strict inclusion criteria for the included studies, so this study can be considered to have made credible findings. Our systematically summarized components of the VR interventions provide insights into further VR intervention development for these populations. The findings show that VR-based interventions have substantial benefits in decreasing preoperative anxiety in patients undergoing elective surgery with anesthesia, especially for the pediatric population via the distraction approach. Future studies should develop tailored VR interventions for different age populations with diverse needs.

The distraction approach should be adopted for pediatric participants, as it offers the chance to reduce preoperative anxiety by relaxing participants with a child-friendly virtual version of the operating theater in which they can become accustomed to the environment and procedures associated with anesthesia and elective surgery [79]. As for the intervention contents, the included studies used diverse VR distraction strategies, with pediatric interventions using immersive environments (eg, animated games and nature exploration) [65,71,76] and adult-focused approaches offering customizable nature scenes paired with auditory relaxation [48,51,58,60,61,63,73,75,77,78]. On the other hand, the VR exposure interventions predominantly featured step-by-step guided tours of surgical procedures, with pediatric studies tailoring narratives through age-adapted versions (eg, cartoon penguin guides [45,54,67]) or gamified adventures [44,59] to indicate perioperative workflows. Seven studies systematically structured storylines from preoperative admission to postoperative recovery. The majority of adult-focused VR exposure interventions also comprehensively simulated the perioperative journey through hospital environments. Notwithstanding, few included studies designed the VR video surrounding the whole perioperative period (ie, postsurgery subprocesses). Regarding the aforementioned duration of the reviewed interventions, the VR-based intervention could have a duration of anywhere between 3 [56] and 21.60 [50] minutes. Additionally, most studies adopted VR HMDs with built-in display units or additional smartphone devices as display units.

With the evolution of VR technology into a smaller, more compact but powerful system, wearable and lightweight VR systems have become useable, accessible, and affordable in health care settings [80,81]. The included studies revealed that consumer-level built-in displays, such as the Oculus Rift device [44], the HTC Vive HMD [24,53,77], and PICO G2 [66,68], or even a smartphone-equipped HMD [45,48,49,51,55,60,63,64,70,71,75,76], are already able to render high-fidelity VR images and can be efficiently applied in clinical settings with promising results. With the consideration of the cost of devices, software development, portability, and device deployment, standalone HMDs, such as the Oculus Go [54,56,69,72,82], other health care professionals in most general clinical settings or smartphone-equipped HMDs, such as the Samsung Gear VR device [45,48,49,51,60,70,75,76,83], would be a desirable option for implementation by nursing teams. This is far-reaching to making evidence-based treatment more accessible to those who are unable (eg, due to mobility or geographic limitations) or unwilling (eg, due to perceived stigmatization) to receive in-person treatment [84]. However, the cost-effectiveness of VR-based interventions was rarely described in detail in the included studies. Therefore, further rigorous RCTs, including economic evaluations, are highly recommended.

The findings of the systematic review and meta-analysis showed that the VR-based distraction approach had a more significant effect size. The results are in line with those of Simonetti et al [37]. This is probably because exposure methods might risk cognitive overload (eg, medical jargon), and reactivating fear memories, whereas the distraction approach may replace negative cognitions with positive immersion (eg, virtual companions). On the other hand, children’s limited attention spans make distraction-based approaches effective, as these methods rely on intuitive emotional experiences (eg, novelty and playful engagement) rather than complex cognitive processing (eg, understanding surgical terminology), thereby aligning with their underdeveloped mentalization capacities [37]. However, current evidence remained limited by small samples and short-term follow-ups, necessitating multicenter RCTs to confirm sustained benefits (ie, postoperative adherence and anxiety-driven care avoidance).

Moreover, although there is no gold standard for measuring preoperative anxiety and different measuring tools were adopted in the included studies, the meta-analysis suggested that the VR-based interventions sustained a more significant effect size for children than for adults. This result echoed that of a meta-analysis of preoperative anxiety management through VR-based interventions in the pediatric population [54]. However, to our knowledge, there were no reports of meta-analyses on adult and child populations for comparison. To gain a more comprehensive understanding of the topic and inform the development of an effective intervention, this review made a deliberate effort to include research conducted on child populations. The potential reasons why preoperative VR interventions may be more effective in children compared to adults are that first, children’s immature prefrontal cortex might heighten responsiveness to multisensory VR stimuli (eg, dynamic games), which could disrupt negative emotional processing by monopolizing attention, whereas adults’ reliance on endogenous cognitive regulation may limit exogenous VR efficacy despite structured informational exposure [85]. In addition, enhanced pediatric effects may also reflect greater autonomic sensitivity to immersive stimuli and intrinsic tech affinity, contrasting with adults’ pragmatic tool expectations and physiological habituation [86]. Future studies should explore optimal timing and frequency for VR to maximize effectiveness across different age groups.

### Limitations

The limitations of this systematic review and meta-analysis should be noted. First, since the means and SDs of 6 studies [24,30,44,45,49] were calculated from median values and IQRs, potential deviation might be reflected in the estimations. Second, considerable heterogeneity was observed in the meta-analysis of the overall effect. This variability could be due to differences in study settings, types of surgery, population characteristics, sample sizes, VR devices and interventions, intervention durations, and measuring instruments. Indeed, there was diversity among the standard usual care in the control groups, such as providing information by iPad [51] and audio-visual descriptions [74], which might have induced variability in the results. However, although the results should be interpreted with notable statistical heterogeneity, the meta-analysis provides information on and reveals insights into the effectiveness of the VR-based intervention for preoperative anxiety management. Third, the specificity of study populations (ie, exclusively elective surgery populations) may restrict the generalizability of conclusions to other clinical scenarios, such as emergency care or populations with diverse chronic illnesses. Moreover, the absence of longitudinal tracking for postoperative long-term behaviors may prevent validation of the sustained benefits of VR-based intervention.

### Implications for Future Studies and Clinical Practice

Preoperative anxiety negatively impacts patients and health care systems. While nonpharmacological interventions like clown and music therapy are preferred, limitations hinder the quality of care for the patient. Advances in IT have led to the promising use of VR in clinical settings for anxiety management [62]. Evidence shows VR effectively reduces preoperative anxiety in both adults and children. Both distraction and exposure approaches are beneficial, with distraction potentially offering greater advantages than the exposure approach [63]. Although the development of VR-based interventions is feasible from technological and economic aspects to the best of our knowledge, more studies are necessary to evaluate the effectiveness of VR-based interventions using the distraction approach to reduce preoperative anxiety in various cultural contexts. It is also of significance to compare the effects of VR distraction interventions with other non–distraction-based VR interventions (ie, exposure) in diverse populations to further consolidate the implications of distraction as a vital medium.

The VR-based intervention, known for its ease of use and low initial cost, can be integrated into routine care for preoperative patients across hospital clusters. Developing a codelivery package based on this review’s findings will facilitate implementation. Additionally, the VR video could be incorporated into existing smartphone apps for preoperative services, allowing for remote access. The widespread availability of entry-level VR headsets supports scalable home deployment. Given the rapid adoption of telemedicine during the COVID-19 pandemic, exploring remote modalities for VR-based interventions in managing preoperative anxiety is important in the postpandemic era.

Additionally, some patients, particularly older or underserved patients, face economic barriers and limited access to VR technology for preoperative anxiety management. To enhance accessibility and affordability, device lending programs could be established in partnership with local health care organizations, community centers, charities, hospitals, and clinics to provide access for those without equipment. Furthermore, the researchers could implement tiered pricing or payment models based on income, and they could collaborate with payers for coverage to further facilitate access. Additionally, providing technical support, user-friendly tutorials, and leveraging telehealth will improve usability. Collaborating with community organizations to create VR access points and developing culturally sensitive, multilingual content will ensure that diverse populations from diverse cultural and socioeconomic backgrounds can benefit from these interventions.

### Conclusions

VR-based interventions for managing preoperative anxiety may be a novel and effective approach that can be further developed as an innovative method of enhancing the quality of patient care. However, the conclusions of this review should be interpreted with caution due to methodological heterogeneity of the included reviews. High-quality RCTs that focus on specific age groups and use a tailored approach, devices, and validated instruments are needed to further confirm the effects of VR in clinical practice.

## Data Availability

All data generated or analyzed during this study are included in this published article and its supplementary information files.

## Appendix

Multimedia Appendix 1 PRISMA checklist.

Multimedia Appendix 2 Search strategy with search terms.

Multimedia Appendix 3 Characteristics and results of included studies on VR-based interventions for managing preoperative anxiety.

## Abbreviations

HMD: head-mounted display
PRISMA: Preferred Reporting Items for Systematic Reviews and Meta-Analyses
RCT: randomized controlled trial
SMD: standardized mean difference
VR: virtual reality
