# Supplementary material for: Effects of Virtual Reality–Based Interventions on Preoperative Anxiety in Patients Undergoing Elective Surgery With Anesthesia: Systematic Review and Meta-Analysis
Source: J Med Internet Res. 2025 Apr 30;27:e55291. doi: 10.2196/55291 (PMC12079079; doi:10.2196/55291)
Supplement: Multimedia Appendix 2 [file jmir_v27i1e55291_app2.docx]

The search terms of both text words and Medical Subject Heading terms, with a combination of wildcards and truncations of ‘anxiety’, ‘preoperative’, and ‘VR’ were used as shown below:

Terms related to ANXIETY:

(anxiet* or afraid or nervous* or worr* or fear* or panic* or distress* or emotional adj3 stress or psychologic* adj3 stress or anxious or (feel* adj3 (apprehens* or dread or terror*)) or 焦慮 or 憂慮)

Terms related to PREOPERATIVE:

(surgical procedure* OR operati* or preoperative care* or preoperative-care* or surger* or surgical* or preop* or pre-op* or periop* or peri-op* or preoperat* or 術前 or 手術前 or 手術準備 or 預備手術)

Terms related to VR:

(Virtual realit* or virtual-realit* or VR or 虛擬實景 or 虛擬現實)

**A sample searching strategy in Medline**

| **Database** | **Searching strategy** |
| --- | --- |
| **Medline** | 1 (VR or ((simulat* or virtual* or artificial*) adj3 realit*) or simulat*).mp.  2 exp Virtual Reality/  3 exp Virtual Reality Exposure Therapy/  4 1 or 2 or 3  5 exp Anxiety/  6 (anxiet* or afraid or nervous* or worr* or fear* or panic* or distress* or emotional adj3 stress or psychologic* adj3 stress or anxious or (feel* adj3 (apprehens* or dread or terror*)).mp.  **7** 5 or 6  8 exp Preoperative Period/ or exp Preoperative Care/  9 (surger* or surgi* or operat*).mp.  10 An?esthesia.mp.  11 exp Anesthesia/  12 10 or 11  13 8 and 9 and 12  14 4 and 7 and 13 |
