# Supplementary material for: Effects of Virtual Reality–Based Interventions on Preoperative Anxiety in Patients Undergoing Elective Surgery With Anesthesia: Systematic Review and Meta-Analysis
Source: J Med Internet Res. 2025 Apr 30;27:e55291. doi: 10.2196/55291 (PMC12079079; doi:10.2196/55291)
Supplement: Multimedia Appendix 3 [file jmir_v27i1e55291_app3.docx]

**Multimedia Appendix 3**

Table 1 Characteristics and Results of Included Studies on VR-Based Interventions for Managing Preoperative Anxiety

| **Study** | **Origin of study / Type of surgery** | **Sample size**  **Gender**  **Population: Mean age** | **VR devices** | **Approach and treatment intervention** | **Control** | **Intervention duration** | **Measurement tool for preoperative anxiety** | **Key findings** |
| --- | --- | --- | --- | --- | --- | --- | --- | --- |
| Abbasnia 2023 [63] | Iran / Laparoscopic Cholecystectomy | n = 145  M: 22, F:123  Adult:  43.9 years | Unnamed VR eyeglass | Distraction: One of the  three 360-degree images of nature, space, and the ocean  Education: Watch the first animation-simulated film of pre-operative education and listen to  the sound through headphones | CAU: Routine care of the ward | 5 minutes | The State-Trait Anxiety Inventory (STAI) | Significant less pre-operative anxiety in the two VR groups, compared with the CAU |
| Akar 2024 [48] | Turkey / open-heart surgery | n = 90  M: 62, F: 28  Adult:  62.8 years | Samsung-branded,  compatible with smartphones and has an Android operating system | Distraction: Virtual Nature 360°- 5.7K Nature Meditation for Oculus Quest | CAU: Nurse visited patients to facilitate their transfer to the waiting room of the operating theater | 6.10 minutes | Visual Analog Scale-Anxiety (VAS-A) | No significant difference between the anxiety levels of the study groups |
| Amiri 2023 [64] | Iran / open-heart surgery | n = 60  M: 38, F: 22  Adult:  56.4 years | Unnamed brand VR | Exposure: VE video of the surgery process | CAU: Conventional video intervention | 4 minutes and 35 seconds | STAI | No statistically significant difference between the groups |
| Bekelis 2017 [74] | US / Cranial and spine procedure | n = 127  M: 74, F:53  Adult:  55.3 years | Oculus VR device | Exposure: VR video describing the preoperative and postoperative experience of the day of the surgery | CAU: Routine audio-visual description of preoperative experience | 5 minutes | Amsterdam Preoperative Anxiety and Information (APAIS) | Less anxiety in interventional group than in CAU |
| Buyuk 2021 [71] | Turkey / Circumcision | n = 78  M: 78  Children:  6.6 years | Unnamed VR eyeglass | Distraction: Watched one of two VR programs (Amazon forests, water skiing) | CAU: Clinical routine care | 4.5 minutes | Children’s anxiety meter scale  (CAM-S) | Less anxiety in interventional group than in CAU |
| Carbό 2024 [70] | Spain / elective low-complexity surgery requiring general anaesthesia with inhalation induction | n = 241  M: 186, F: 55  Children:  6.4 years | Samsung Gear VR, Samsung  Electronics, Seoul, South Korea | Exposure: VR-based educational video on the surgical  process | CAU: Providing the usual information consisting of a verbal and written account of the entire surgical process | 5 minutes | The modified Yale Preoperative Anxiety Scale (mYPAS) | Less anxiety in interventional group than in CAU |
| Chiu 2023 [62] | Hong Kong / first elective surgery procedure under general anesthesia | n = 74  M: 38, F: 36  Adult:  46.3 years | Oculus Quest 2;  Meta | Exposure: VR video included a virtual tour simulating the entire journey of the perioperative process | CAU: Standard care in the preanesthetic assessment clinic |  | APAIS | Less preoperative anxiety in interventional group than in CAU |
| Dehghan 2019 [30] | Iran / Abdominal surgery | n = 40  M:31, F:9  Children:  7.35 years | Unnamed VR eyeglass with a built-in display unit | Exposure: VR video of the operating theatre using VR to present the simulated steps of going to the operating theatre | CAU: Parents of patients touch and care for their children | 5 minutes | Yale Preoperative Anxiety Scale questionnaire (YPAS) | Less anxiety in interventional group than in CAU; the subscale results was significant for activity, vocalization, emotional expressivity and non-significant for the state of apparent arousal |
| Eijlers 2019 [24] | Netherlands / Maxillofacial, dental or  ear-nose-throat (ENT) daycare surgery | n = 191  M:101, F:90  Children:  7.89 years | HTC Vive (HTC Corporation,  Xindian, New Taipei, Taiwan) HMD | Exposure: Computer-generated VR environment of the operating theatre and medical staff | CAU: Usual care | Approximately 15 minutes | ~~Modified Yale~~  ~~Preoperative Anxiety Scale (~~mYPAS~~)~~ | Non-significant difference in anxiety between the interventional and control groups at both hospital admission, holding area and induction of anesthesia |
| El Mathari 2024 [68] | Netherlands / cardiac surgery | n = 121  M: 98, F: 23  Adult:  67.5 years | Pico G2 4K VR headset | Exposure: immersive educational 360◦ VR tour | CAU: Traditional patient education | 14 minutes | STAI  APAIS | Neither STAI nor APAIS scores showed differences in preoperative anxiety between both groups |
| Esposito 2022 [65] | Italy / elective surgery under general anesthesia | n = 40  M: 25, F: 15  Children:  14.5 years | HMD integrated with an earphones  system and touch controllers | Distraction: Watching a 360◦ movie chosen by the patient | CAU: no VR experience | 5 minutes | Facial affective scale (FAS) | The very relaxed/relaxed face selection rate using FAS was significantly higher in intervention group than in CAU |
| Grab 2023 [50] | Germany / coronary artery bypass graft  surgery, surgical aortic valve replacement or  thoracic aortic aneurysm repair or a combination thereof | n = 99  M: 87, F: 12  Adult:  65.0 years | Oculus Quest 2, Meta  Platforms Inc., Menlo Park, CA, USA | Exposure: VR educational model | CAU: standardized pre-printed paper-based models;  3D-printed model | 21.6 minutes | German short version of the STAI  VAS | A signifificant reduction in anxiety measured by Visual Analog Scale was  achieved after patient education with virtual reality models |
| Hendricks 2020 [75] | US / first-time sternotomy | n = 20  M: 18, F: 2  Adult:  66.5 years | Samsung Gear Occulus and audio  headset (Ridgefield Park, NJ) fitted with a Samsung Galaxy S7 device to deliver VR content | Distraction: Played a nonviolent  VR game titled “Bear Blast” | CAU: A tablet-based game | 20 minutes | STAI | Superior and significant improvements in feeling calm and significant reductions in feeling tense and strained in the VR group than in CAU |
| Jung 2021 [76] | US / elective surgery or procedure  requiring general anesthesia via inhaled induction | n = 70  M: 34, F: 36  Children:  8.0 years | Customized Samsung Gear VR headset (Samsung  Electronics, Suwon, South Korea) | Distraction: preselected, interactive game designed for pediatric perioperative  featuring an animated animal character moving through a landscape | CAU: no VR experience | Unclear | mYPAS | Less preoperative anxiety in interventional group than in CAU |
| Kwon 2023 [66] | Korea / elective surgery under general anesthesia | n = 80  M: 52, F: 28  Adult:  41.8 years | PICO G2 model (PICO Interactive Korea Co.,  Ltd., Seoul, South Korea) | Exposure: VR surgery experience education | CAU: conventional verbal education | 11 minutes | APAIS | Less preoperative anxiety in interventional group than in CAU |
| Lahti 2020 [60] | Finland / Dental treatment | n = 255  M:84, F:171  Adult:  52.5 years | Samsung Gear VR with a smartphone (Samsung Galaxy S7) | Distraction: 1 out of 5 360° videos immersed in a peaceful virtual landscape (beach, waterfall, underwater, space float, paddling) | CAU: Sitting in small alcoves with a seat and a table for 3 min with no intervention | 1 to 3.5 minutes | Modified Dental  Anxiety Scale (MDAS) | Less anxiety in interventional group than in CAU; subgroup analysis showed significant results in females but not in males |
| Larsson 2023 [61] | France / Invasive coronary angiography | n = 156  M: 111, F: 45  Adult:  62.6 years | Unnamed brand VR headset and audio headphones | Distraction: One of five VR themes (Zen garden, forest, mountain, beach, diving) | CAU: Usual care | 20 minutes | Visual Analogue Scale for Anxiety (VASA) | Non-significant difference in anxiety between the groups |
| Liu 2023 [57] | China / carotid  artery stenting | n = 107  M: 87, F: 20  Adult:  65.0 years | YVR2 (Yuweia  Technology Corp, Shanghai, China) | Exposure + distraction: Introduction to the surgery; Patient interview; Scenic tour | CAU: Psychological care and watch a video of the intervention on iPad Air | 18 minutes | STAI | Less preoperative anxiety in interventional group than in control group |
| Noben 2019 [55] | Netherlands / Caesarean delivery (CD) | n = 97  F:97  Adult:  32.86 years | Unnamed brand Smartphone HMD | Exposure: 360° VR video showing all the aspects of a CD | CAU: Provide standard preoperative information | 385 seconds | Visual Analogue Scale (VAS) | Non-significant difference in anxiety between interventional and control group for patients and their partners |
| Pool 2022 [69] | Netherlands / transcutaneous patent foramen ovale or atrial septal defect closure | n = 50  M: 24, F: 26  Adult:  43.8 years | Oculus GO headsets (Oculus, Facebook Technologies, LLC, Menlo Park,  CA, USA) | Exposure: 360° VR video | CAU: Routine oral information on  the procedure | 5 minutes | STAI  APAIS | During follow-up, anxiety (STAI) increased in the control group, but remained unchanged in the intervention group. No differences were found for the APAIS anxiety scale. |
| Park 2019 [67] | Korea / elective surgery under general anesthesia | n = 80  M: 47, F: 33  Children:  6.9 years | Smart Mirroring 2.0 SE; SK telecom, Seoul, Korea | Exposure: VR video: Poroto the little penguin introduced the detailed preoperative preparation process | Watched a  VR-guided tour of the operating theatre via a smartphone | 4 minutes | validated Korean version of m-YPAS | Less preoperative anxiety of children in interventional group than in control group |
| Prabhu 2024 [77] | US / Total Knee Arthroplasty | n = 30  M: 7, F: 23  Adult:  66.3 years | A tethered HTC Vive Pro HMD | Distraction: VR natural environment with biofeedback | CAU: relax without any specific instructions or strategies  2D video: virtual environment via laptop | 10 minutes | VAS  STAI | VR significantly decreased anxiety post-intervention compared with the control group |
| Robertson 2017 [51] | Australia / Arthroscopic knee surgery | n = 60  M:38, F:22  Adult:  47 years | Samsung Gear VR with smartphone (Samsung Note 4) | Distraction: VR immersion of ‘Perfect Beach’ scenario | CAU: Standard hospital care  iPad group: Watched videos of 'Beautiful beaches from around the world'  for 9 minutes with the same narrated audio | 9 minutes | Hospital Anxiety and Depression Scale (HADS) | Marginal difference in anxiety between interventional (VR) and control group (*P* = 0.055). There was no significant difference between the VR and iPad groups |
| Ryu 2017 [49] | South Korea / General anesthesia and elective surgery | n = 69  M:41, F:28  Children:  6 years | Samsung VR Gear with smartphone (Galaxy S6) | Exposure: 360° VR video with children-friendly characters describing the preoperative experience | CAU: Standard information | 4 minutes | mYPAS | Significant less anxiety in interventional group than in CAU group; significant difference in interventional group based on Induction Compliance Checklist and procedural behaviour rating scale (PBRS) scores |
| Ryu 2018 [44] | South Korea / General anesthesia and elective day surgery | n = 69  M:40, F:29  Children:  5.5 years | Oculus Rift (Oculus VR, Menlo Park, CA, USA), and a hand and finger motion controller, Leap Motion Controller (Leap Motion, San Francisco, CA, USA) | Exposure: 360° VR game experience of the preoperative process and general anesthesia induction | CAU: Conventional mode of education | 5 min | mYPAS | Less anxiety in interventional group than in the CAU group; the significant difference in interventional group based on Induction Compliance Checklist and PBRS scores; comparable postoperative behaviour disturbance by Post-Hospitalization Behaviour Questionnaire for Ambulatory Surgery |
| Ryu 2019 [45] | South Korea / Genera anesthesia and elective surgery | n = 80  M:50, F:30  Children:  6 years | Samsung VR Gear with smartphone (Galaxy S6) | Exposure: 360° VR video with children-friendly characters describing the preoperative experience | CAU: Standard information | 4 minutes | mYPAS | Less anxiety in interventional group than in the CAU group; non-significant in the incidence of emergency delirium |
| Ryu 2022 [54] | South Korea / General anesthesia and elective surgery | n = 105  M: 48, F: 57  Children:  5.3 years | Oculus Go (Oculus VR) | Exposure A: VR tour on the process of anesthesia at the outpatient clinic when the operation was decided  Exposure B: VR tour in the reception area of the operating theater 10min before anesthesia | CAU: Received standard information concerning the  process of anesthesia and operation 10 min before anesthesia | 4 minutes | mYPAS | Significantly less anxiety in VR B group than that of the control and VR A groups |
| Schmid 2024 [56] | Australia / Elective surgery | n = 67  F: 67  Adult:  57.0 years | Oculus Go® from Meta Reality Labs, Menlo Park, CA, USA | Exposure: VR environment of the surgery process | CAU: Usual care | 3 minutes and 34 seconds | VAS | Less anxiety in interventional group than in CAU |
| Subramaniam 2025 [78] | US / first-time sternotomy or thoracotomy | n = 100  M: 55, F: 45  Adult:  60.5 years | Unnamed brand VR | Distraction: Virtual environments which were reflective of the four seasons | CAU: A non-immersive tablet interface | 10 minutes | STAI | Less overall state anxiety in VR group than in CAU |
| Turgut 2024 [72] | Turkey / First-time surgery | n = 70  M: 52, F: 18  Children:  6.9 years | Oculus Go VR | Exposure: 360-degree tour video of the preoperative and postoperative processes | CAU: Standard preoperative educational procedure | Unclear | Children’s  State Anxiety Scale | Less preoperative anxiety in interventional group than in control group |
| Turrado 2021 [52] | Spain / Elective surgery for newly diagnosed with colorectal cancer | n = 126  M: 76 F: 50  Adult:  66.0 years | Bluebee™ Genuine VR 3D Glasses | Exposure: VR environment of the perioperative process | CAU: no VR exposure | 16 minutes and 34 seconds | STAI-S  HADS | Less preoperative anxiety in interventional group than in control group |
| Ugras 2023 [73] | Turkey / colorectal  and abdominal wall surgery | n = 86  M: 51 F: 35  Adult:  43.9 years | VR BOX 2 | Distraction: five  three-dimensional videos (underwater world, museum trips, a  walk in the forest and park, beach trips, and space travel) with  relaxing music | CAU: routine preoperative procedure | 10 minutes | The Anxiety Specific to Surgery Questionnaire (ASSQ) | Less preoperative anxiety in interventional group than in control group |
| Wang 2024 [58] | China / elective gynecology laparoscopy | n = 115  F: 115  Adult:  37.9 years | Oculus Quest 2 VR Virtual Reality  Headset™ (Headset Model Number: KW49CM, Meta, USA) | Distraction: VR film featuring natural landscapes | CAU: standard preoperative care | 15 minutes | HADS | Less preoperative anxiety in interventional group than in control group |
| Wu 2022 [59] | China / First operation with general anesthesia | n = 99  M: 86, F: 13  Children:  7.5 years | CV1 PRO, NOLO  VR, Beijing, China | Exposure: VR video with children-friendly characters describing the surgery process | CAU: Conventional preoperative preparation | 5 minutes | The modified Yale Preoperative  Anxiety Scale-Short Form (mYPAS-SF) | Lower mYPAS-SF scores when leaving the waiting area and during anesthesia induction in VR group than in CAU |
| Yang 2019 [53] | South Korea / Arthroscopic knee surgery | n=48  M:30 F:18  Adult:  35.25 years | HTC Vive HMD | Exposure: Watch a 3D model of your own MRI knee image | CAU: Information about their MRI | Varies | APAIS | Less anxiety in interventional group than in CAU; the subscale showed non-significant in anesthesia-related anxiety (sum A); information-need component (sum I); but significant in surgery-related anxiety (sum S) and combined anxiety component (sum C) |

Abbreviations: CAU = Care as usual; HMD: head-mounted display; MRI = Magnetic Resonance Imaging; VR = Virtual realit
